# Coronavirus Disease 2019–Related Stigma in China: A Descriptive Study

**DOI:** 10.3389/fpsyg.2021.694988

**Published:** 2021-08-11

**Authors:** Li Zhao, Zhen Wang, Jian Guan, Panyan Shen, Wen Zhao, Guoguo Zuo

**Affiliations:** ^1^Department of Social Psychology, Zhou Enlai School of Government, Nankai University, Tianjin, China; ^2^Department of Applied Psychology, School of Law, Southwest University of Science and Technology, Sichuan, China

**Keywords:** COVID-19, stigma, knowledge, China, descriptive study

## Abstract

Coronavirus disease 2019 (COVID-19) tremendously impacts the physical and mental health of humans worldwide. Consequently, studies on COVID-19 remain extensive. However, most of them were mainly focused on the pathological mechanisms and treatment methods from medical perspectives. Various reports have indicated that COVID-19 is closely related to stigma and discrimination, but little statistical information has been integrated quantitatively to describe the situation in China. Thus, this study investigated the COVID-19-related stigma of individuals. We collected the online survey data from 1,920 Chinese participants from October to December 2020. Findings showed that 306 (15.94%), 285 (14.84%), 265 (13.80%), and 100 (5.21%) participants endorsed stigma toward individuals in high-risk areas, recovered patients with COVID-19, families of recovered patients with COVID-19, and frontline healthcare providers, respectively. To understand the possible factors that could impact the COVID-19-related stigma, knowledge about COVID-19 was investigated. Generally, knowledge about COVID-19 was negatively associated with COVID-19-related stigma in general, while no significant relationship existed between the knowledge about COVID-19 and the COVID-19-related stigma in the groups who had held COVID-19-related stigma. Ultimately, individuals showed COVID-19-related stigma toward recovered patients and their families, individuals in high-risk areas, and frontline healthcare providers to some extent. The results of this study can provide reference to nations, governments, and organizations in addressing the stigma issues raised by the COVID-19 pandemic.

## Introduction

Historically, infectious diseases posed a huge threat to the lives of people (Ackerman et al., 2018). Even at present, deaths from infectious diseases account for roughly one-quarter worldwide (World Health Organization, 2015). To mitigate the threat caused by various diseases, people have evolved the behavior immune system (BIS) (Schaller and Park, 2011; Taylor, 2019). The activation of the BIS has implications for the affective, cognitive, and behavioral responses of people at individual and group levels, such as devoting more visual attention to cues related to diseases (Ackerman et al., 2009; Stone and Potton, 2019), increasing intentions to use condoms in sexual attitudes (Tybur et al., 2011), producing more prejudices against out-groups (Kusche and Barker, 2019), and adopting more socially conservative values (Tybur et al., 2016).

Among these studies, one of the most intriguing and well-studied results of the BIS may be the stigma toward individuals possessing cues related to infectious disease (Ackerman et al., 2018). The BIS has implications for prejudices against individuals who have diseases, such as HIV/AIDS, severe acute respiratory syndrome (SARS), and influenza A virus (H1N1) (Kurzban and Leary, 2001; Oaten et al., 2011; Murray and Schaller, 2016). Specifically, due to the BIS, individuals may—in facing cues related to diseases—trigger disgust, worry, and anger in affective responses, vulnerability beliefs and group stereotypes in cognitive responses, and avoidance and protection in behavioral responses (Ackerman et al., 2018). Therefore, coronavirus disease 2019 (COVID-19), as a novel infectious disease, may be stigmatized due to the protective function of psychological mechanisms of the BIS. Thus, in this study, whether there exists COVID-19-related stigma in China was investigated.

## Stigma

Stigma refers to a devalued social identity, which is associated with attributes and characteristics of stereotype, prejudice, and discrimination (Crocker et al., 1998). Stigma significantly and negatively affects individuals by decreasing self-esteem, interfering with family relationships, and limiting opportunities in the job market (Zolezzi et al., 2018). Therefore, a growing number of researchers focus on the stigma, such as weight stigma (Papadopoulos and Brennan, 2015; Hackman et al., 2016), homosexuality stigma (Preciado et al., 2013; Lelutiu-Weinberger et al., 2019), and illness stigma (Casados, 2017; Norman et al., 2017; Kosyluk et al., 2018; Caqueo-Urízar et al., 2020).

Among various types of stigmas, illness stigma can be divided into two main types. One is the mental illness stigma, such as schizophrenia, depressive, and anxiety disorders. For example, Wang et al. (2012) used an implicit association test and found that individuals tended to associate mental illness-related words (e.g., “depressive disorder”) with negative words (e.g., “dangerous,” “negative,” “evil”) on cognition, emotion, and behavioral tendency, which implied that individuals had an implicit stigma to mental illness. Additionally, studies on explicit stigma to mental illness also found that individuals held stigmatizing attitudes toward mental illness (Peris et al., 2008; Sandhu et al., 2019). Another type of illness stigma is the psychical illness stigma, particularly related to infectious diseases, such as HIV/AIDS, SARS, and TB (Mak et al., 2006; Williams et al., 2011; Wagner et al., 2017). For example, individuals who suffered from or were suspected of having HIV/AIDS had experienced various discriminations induced by stigma, such as disrespect, rejection, and being ignored by families, friends, or strangers (Crandall and Coleman, 1992; Herek, 1999). In 2003, SARS had spread to over 29 countries globally, and it was also associated with various forms of stigma, such as being shunned, insulted, marginalized, and excluded from society (Lee et al., 2005).

## Coronavirus Disease 2019–Related Stigma

In recent decades, an increasing body of evidence has indicated that infectious diseases related to stigma have caused many serious social problems, with the emergence of a growing number of novel infectious diseases worldwide (Williams et al., 2011; Dubey et al., 2020). COVID-19 is a novel infectious disease that broke out in Wuhan, China in December 2019. Since then, COVID-19 swept all provinces of China and spread rapidly across the globe. According to the WHO, as of May 27, 2021, there have been 108,361 confirmed cases, with 4,881 deaths, in China. Even worse, 167,492,769 confirmed cases with 3,482,907 deaths have been reported globally (World Health Organization, 2021). To decelerate the spread of COVID-19, in addition to health organizations and governments recommending protective measures, the BIS of individuals triggers their various affective, cognitive, and behavioral responses to protect themselves (Makhanova and Shepherd, 2020). For example, that diseases related to disgust and avoidance behavior were positively associated with COVID-19 concern, which means that the more people perceived COVID-19 serious, the greater disgust they might feel toward COVID-19 and its related cues, the greater avoidance of touching others (Makhanova and Shepherd, 2020; Shook et al., 2020). These negative effects and behaviors conformed to the manifestations of stigma (Link and Phelan, 2001). In this case, as one of the most infectious diseases in history (Upadhyay et al., 2020), we assumed that COVID-19-related stigma exists.

However, as far as we know, few studies have investigated the COVID-19-related stigma based on an empirical study. Those few theoretical studies reported that many individuals, such as frontline healthcare providers and individuals who were living in high-risk zones, might experience negative attitudes caused by COVID-19 (Adja et al., 2020; Singh and Subedi, 2020). Additionally, these individuals suffered different kinds of discrimination, such as isolation, refusal of service, harassment, and bullying (Turner-Musa et al., 2020). Given these negative consequences caused by COVID-19-related stigma, individuals who have or are suspected of COVID-19 infection tend to lag in seeking medical care or even hide their illness, which will threaten the safety of others and increase the difficulty in containing the epidemic (Dubey et al., 2020). Therefore, this study aims to investigate whether and to what extent the COVID-19-related stigma exists in China to provide evidence for the interventions of COVID-19-related stigma and further help control the spread.

We hypothesized that COVID-19-related stigma exists in China. Specifically, individuals endorse stigma toward recovered patients with COVID-19, their families, friends, neighbors, and frontline healthcare providers, and individuals in high-risk areas (H_1_). Moreover, stigma related to infectious diseases was negatively associated with infectious diseases-related knowledge (Balfour et al., 2010; Farotimi et al., 2015; Fischer et al., 2019). Therefore, a negative relationship between the COVID-19-related stigma and the knowledge about COVID-19 exists (H_2_).

## Materials and Methods

### Participants and Procedures

The Chinese professional survey website Wenjuanxing (www.wjx.cn, which is similar to SurveyMonkey) was used to collect the data. In this study, a survey, which contained the questionnaire (see details in the “Measures” section), was built on Wenjuanxing and a link was created for it. Then, the link to the survey, accompanied by a brief introduction, was distributed to three participant pools *via* two social network sites, namely, WeChat and QQ (which are similar to Twitter). If individuals were interested in the survey, they could participate through the Wenjuanxing link and help us share the link and introduction of the survey with others if they decide to. Given that this study aimed to describe the situation of COVID-19-related stigma in China, we surveyed as many participants as possible to collect the data. A total of 2,239 participants from 26 provinces in China participated in the survey. The exclusion criteria were as follows: (1) under 16 years of age; (2) response time was <3 min; (3) there were missing data; and (4) response regularly, which means participants made the same choice for all items. Resultantly, 319 questionnaires were removed, leaving 1,920 questionnaires in the final analyses. The mean age of the sample was 20.51 years (*SD* = 4.51 years) and ranged from 16 to 54 years. The detailed demographic characteristics are presented in Table 1.

**Table 1:** Demographic characteristics of the sample (*N* = 1,920).

**Demographic variables**		***N***	**%**
Gender	Male	827	43.07
	Female	1,093	56.93
Education level	High school and below	25	1.30
	College	309	16.09
	Bachelor's degree	1,495	77.87
	Master's degree and above	91	4.74
Age (years)	16–20	1,452	75.63
	21–30	379	19.74
	31–40	73	3.80
	41–54	16	0.83
Social class	1	42	2.19
	2	88	4.58
	3	243	12.66
	4	322	16.77
	5	602	31.35
	6	350	18.23
	7	176	9.17
	8	47	2.45
	9	12	0.62
	10	38	1.98

### Measures

The questionnaire used in this study involved three sections:

#### Demographic Information

Participants were asked to fill in the information regarding their age, gender, hometown province, education level, and subjective social class. The subjective social class was measured using the MacArthur Scale of Subjective Socioeconomic Status (Adler et al., 2000). This scale is a ladder that has 10 rungs. Each rung represents a different level of social class. Participants were told that the bottom of the ladder represents the lowest social class, which means their income, degree of education, and occupation are at the lowest level, and the top of the ladder represents the highest social class, which means their income, degree of education, and occupation are at the highest level. Based on their true social classes, participants were asked to indicate which level of the ladder they belong to.

#### Coronavirus Disease 2019–Related Stigma

To measure the COVID-19-related stigma, a one-dimensional stigma scale was adopted following the study of Mak et al. (2006). It contains 14 items to assess the COVID-19-related stigma of participants from the following three aspects: affective response (5 items, e.g., “recovered COVID-19 patients are a nuisance” if it is to report the impression to recovered patients with COVID-19. The following examples of items used the same group.), cognitive response (3 items, e.g., “It is only normal that recovered COVID-19 patients are being discriminated against by other people”), and behavioral intention (6 items, e.g., “I will try to keep my distance with recovered COVID-19 patients as much as possible”). Specifically, there were 7 sub-questions corresponding to the 7 target groups under each item in the survey. Moreover, each sub-question had a 6-point scale ranging from 1 (strongly disagree) to 6 (strongly agree). For example, one of the items in the survey is that “I will try to keep my distance with the following groups as much as possible.” Under this item, participants reported their impression toward each target group (7 times in total) on the 6-point scale. After the participants finished the survey, the stigma of participants toward each target group was calculated separately based on the calculation used by Mak et al. (2006). Specifically, after reversing appropriate items (i.e., items 6, 9, 10, 13, and 14), the stigma scores were defined as the mean score of all 14 items, and a higher score represented a higher level of stigma related to COVID-19.

Ultimately, not only infected individuals, but also their families, friends, and neighbors, frontline healthcare providers, and individuals in high-risk areas were vulnerable to stigma (Adja et al., 2020; Singh and Subedi, 2020). To investigate the COVID-19-related stigma, stigma toward these six groups was measured in this study. Additionally, stigma toward individuals who were not related to COVID-19 was measured as the control group. In this study, individuals not related to COVID-19 refer to healthy individuals who were not infected with COVID-19 and were not families, friends, or neighbors of patients with COVID-19. Therefore, a total of seven groups were investigated in this study. In the study of Mak et al. (2006), the values of Cronbach's α of the stigma scale were 0.85, 0.81, and 0.83 for HIV/AIDS, SARS, and TB, respectively. In this study, the internal consistency coefficient of the whole scale was good (Cronbach's α = 0.96). Specifically, for the recovered patients with COVID-19, families of recovered patients with COVID-19, neighbors of recovered patients with COVID-19, friends of recovered patients with COVID-19, frontline healthcare providers, individuals in high-risk areas, and individuals not related to COVID-19, the values of Cronbach's α were 0.80, 0.79, 0.80, 0.80, 0.77, 0.78, and 0.76, respectively.

#### Knowledge About COVID-19

Two items scored on a 10-point Likert scale from 1 (strongly unfamiliar) to 10 (strongly familiar) were adopted from the study of Khasawneh et al. (2020) to assess the degree of the knowledge of participants about COVID-19 (i.e., “the knowledge of potential sources of transmission of COVID-19” and “the knowledge of potential risk factors and virulence of COVID-19”).

## Results

### Descriptive Results of the COVID-19-Related Stigma Scores

The descriptive statistics on stigma toward COVID-19, such as recovered patients with COVID-19, families of recovered patients with COVID-19, neighbors of recovered patients with COVID-19, friends of recovered patients with COVID-19, frontline healthcare providers, individuals in high-risk areas, and individuals not related to COVID-19, are shown in Table 2. The same criterion (i.e., score > 3), as used in the study of Mak et al. (2006), was adopted to indicate that participants endorsed stigmatizing perceptions toward target groups. Although the average stigma scores of participants for these seven groups were all <3 in this study, 306 (15.94%), 285 (14.84%), and 265 (13.80%) participants endorsed stigma (score > 3) toward individuals in high-risk areas, recovered patients with COVID-19, and families of recovered patients with COVID-19, respectively. Additionally, only 120 (6.25%) and 100 (5.21%) participants had stigmatizing perceptions toward individuals not related to COVID-19 and frontline healthcare providers, respectively.

**Table 2:** Descriptive results of the coronavirus disease 2019 (COVID-19)-related stigma scores (*N* = 1,920).

**Variables**	***M***	***SD***	***N*** **(%)**			
	**1 ≤ score ≤ 2**	**2 < score ≤ 3**	**3 < score ≤ 4**	**4 < score ≤ 5**
Recovered patients	2.35	0.64	644 (33.54%)	991 (51.61%)	268 (13.96%)	17 (0.89%)
Recovered patients' families	2.31	0.63	691 (35.99%)	964 (50.21%)	251 (13.07%)	14 (0.73%)
Recovered patients' neighbors	2.28	0.63	726 (37.81%)	948 (49.38%)	234 (12.19%)	12 (0.62%)
Recovered patients' friends	2.26	0.64	757 (39.43%)	925 (48.18%)	226 (11.77%)	12 (0.62%)
Frontline healthcare providers	2.03	0.60	1,021 (53.18%)	779 (40.57%)	118 (6.15%)	2 (0.10%)
Individuals in high-risk areas	2.38	0.65	626 (32.60%)	988 (51.46%)	286 (14.90%)	20 (1.04%)
Individuals not related to COVID-19	1.97	0.58	1,106 (57.60%)	714 (37.19%)	98 (5.11%)	2 (0.10%)

*Score means the stigma score of participants. The COVID-19-related stigma was measured on a 6-point scale*.

### Correlations Among COVID-19-Related Stigma Scores

The correlations among COVID-19-related stigmas to the seven groups are presented in Table 3. The stigma scores of these seven groups, such as recovered patients with COVID-19, families of recovered patients with COVID-19, neighbors of recovered patients with COVID-19, friends of recovered patients with COVID-19, frontline healthcare providers, individuals in high-risk areas, and individuals not related to COVID-19, had significantly positive relationships with one another (*r* ranges from 0.65 to 0.99).

**Table 3:** Correlations between different types of COVID-19-related stigma (*N* = 1,920).

**Variables**	**1**	**2**	**3**	**4**	**5**	**6**	**7**
1. Recovered patients	–	[0.943,0.968]	[0.914,0.944]	[0.900,0.933]	[0.735,0.784]	[0.737,0.793]	[0.661,0.723]
2. Recovered patients' families	0.96^***^	–	[0.970,0.982]	[0.960,0.974]	[0.764,0.811]	[0.768,0.817]	[0.693,0.756]
3. Recovered patients' neighbors	0.93^***^	0.98^***^	–	[0.983,0.989]	[0.776,0.821]	[0.772,0.821]	[0.724,0.783]
4. Recovered patients' friends	0.92^***^	0.97^***^	0.99^***^	–	[0.782,0.827]	[0.771,0.822]	[0.733,0.792]
5. Frontline healthcare providers	0.76^***^	0.79^***^	0.80^***^	0.81^***^	–	[0.684,0.743]	[0.793,0.837]
6. Individuals in high-risk areas	0.77^***^	0.79^***^	0.80^***^	0.80^***^	0.71^***^	–	[0.618,0.684]
7. Individuals not related to COVID-19	0.69^***^	0.73^***^	0.76^***^	0.76^***^	0.82^***^	0.65^***^	–

*** *p < 0.001*.

### Knowledge and COVID-19-Related Stigma

The descriptive analysis of the knowledge of participants about (i.e., sources and risk factors) COVID-19 showed that the level of knowledge among participants toward possible sources of COVID-19 transmission (*M* = 6.79, *SD* = 1.73) and potential risk factors for COVID-19 infection (*M* = 6.91, *SD* = 1.78) was not high. The distributions of the knowledge about COVID-19 transmission and infection are shown in Table 4.

**Table 4:** Descriptive results of the knowledge about COVID-19 (*N* = 1,920).

**Transmission**	***N***	**%**	**Infection**	***N***	**%**
1	10	0.50	1	9	0.50
2	12	0.60	2	14	0.70
3	27	1.40	3	36	1.90
4	85	4.40	4	80	4.20
5	342	17.80	5	289	15.10
6	326	17.00	6	316	16.50
7	466	24.30	7	465	24.20
8	477	19.60	8	384	20.00
9	110	5.70	9	136	7.10
10	165	8.60	10	191	9.90

*The knowledge about COVID-19 was measured on a 10-point scale*.

The correlations between the knowledge about COVID-19 and the COVID-19-related stigma are presented in Table 5. Based on the whole dataset, the possible sources of COVID-19 transmission and the possible risk factors for COVID-19 infection were negatively associated with the stigma toward COVID-19, such as recovered patients with COVID-19, families of recovered patients with COVID-19, neighbors of recovered patients with COVID-19, friends of recovered patients with COVID-19, frontline healthcare providers, individuals in high-risk areas, and individuals not related to COVID-19 (*r* ranges from −0.12 to −0.15).

**Table 5:** Correlations between the knowledge about COVID-19 and the COVID-19-related stigma (*N* = 1,920).

**Variables**	**Knowledge about COVID-19**	
	**Possible sources of COVID-19 transmission**	**Potential risk factors for COVID-19 infection**
Recovered patients	−0.14^***^	−0.14^***^
Recovered patients' families	−0.14^***^	−0.15^***^
Recovered patients' neighbors	−0.14^***^	−0.14^***^
Recovered patients' friends	−0.14^***^	−0.14^***^
Frontline healthcare providers	−0.12^***^	−0.12^***^
Individuals in high-risk areas	−0.13^***^	−0.14^***^
Individuals not related to COVID-19	−0.13^***^	−0.12^***^

*** *p < 0.001*.

The average stigma scores of participants for these seven groups were all <3 in this study, which implied that participants did not endorse the COVID-19-related stigma from the perspective of the whole data. Therefore, to identify the possible sources of and factors that impact stigma, a series of new datasets was composed based on the original dataset when a stigma score of the participant was >3. The specific sample size for each group is shown in Table 6.

**Table 6:** Correlations between the knowledge about COVID-19 and the COVID-19-related stigma (stigma score > 3).

**Variables**	**Knowledge about COVID-19**	
	**Possible sources of COVID-19 transmission**	**Potential risk factors for COVID-19 infection**
Recovered patients (*n* = 285)	0.02 (*p =* 0.69)	0.02 (*p =* 0.74)
Recovered patients' families (*n* = 265)	0.02 (*p =* 0.65)	0.03 (*p =* 0.60)
Recovered patients' neighbors (*n* = 246)	0.07 (*p =* 0.26)	0.09 (*p =* 0.15)
Recovered patients' friends (*n* = 238)	0.06 (*p =* 0.34)	0.07 (*p =* 0.27)
Frontline healthcare providers (*n* = 120)	0.05 (*p =* 0.57)	0.03 (*p =* 0.74)
Individuals in high-risk areas (*n* = 306)	−0.01 (*p =* 0.94)	−0.01 (*p =* 0.92)
Individuals not related to COVID-19 (*n* = 100)	0.04 (*p =* 0.71)	0.05 (*p =* 0.59)

Based on the series of new datasets, the correlations between the knowledge about COVID-19 and the COVID-19-related stigma are presented in Table 6. The possible sources of COVID-19 transmission and the possible risk factors for COVID-19 infection were not significantly associated with the COVID-19-related stigma.

Given that the results of the whole dataset of stigma differed from those of the data when the stigma scores were >3, the correlations between the knowledge about COVID-19 and the dataset when the stigma scores were ≤3 were analyzed (Table 7). The possible sources of COVID-19 transmission and the possible risk factors for COVID-19 infection were all negatively associated with the COVID-19-related stigma, such as recovered patients with COVID-19, families of recovered patients with COVID-19, neighbors of recovered patients with COVID-19, friends of recovered patients with COVID-19, frontline healthcare providers, individuals in high-risk areas, and individuals not related to COVID-19 (*r* ranges from −0.12 to −0.15).

**Table 7:** Correlations between the knowledge about COVID-19 and the COVID-19-related stigma (stigma score ≤ 3).

**Variables**	**Knowledge about COVID-19**	
	**Possible sources of COVID-19 transmission**	**Potential risk factors for COVID-19 infection**
Recovered patients (*n* = 1,635)	−0.14 (*p* < 0.001)	−0.15 (*p* < 0.001)
Recovered patients' families (*n* = 1,655)	−0.15 (*p* < 0.001)	−0.14 (*p* < 0.001)
Recovered patients' neighbors (*n* = 1,674)	−0.14 (*p* < 0.001)	−0.14 (*p* < 0.001)
Recovered patients' friends (*n* = 1,682)	−0.15 (*p* < 0.001)	−0.15 (*p* < 0.001)
Frontline healthcare providers (*n* = 1,800)	−0.14 (*p* < 0.001)	−0.13 (*p* < 0.001)
Individuals in high-risk areas (*n* = 1,614)	−0.15 (*p* < 0.001)	−0.15 (*p* < 0.001)
Individuals not related to COVID-19 (*n* = 1,820)	−0.13 (*p* < 0.001)	−0.12 (*p* < 0.001)

### Differences of COVID-19-Related Stigma on Demographic Variables

Based on the series of new datasets, the results of analyses on the differences of COVID-19-related stigma on demographic variables are as follows (Table 8): no significant differences existed between men and women on the COVID-19-related stigma, except that men (*M* = 3.43, *SD* = 0.34) endorsed a significantly higher level of stigma toward neighbors of recovered patients with COVID-19 than women (*M* = 3.35, *SD* = 0.27), *t*_(244)_ = 2.19, *p* = 0.03, Cohen's *d* (i.e., the effect size) = 0.26, which was small based on the Cohen's conventions (Cohen, 1988). No significant differences existed among different ages on the COVID-19-related stigma. No significant differences existed among different education levels on the COVID-19-related stigma, except that significant differences existed among different educations on stigma toward recovered patients with COVID-19, *F*_(3,281)_ = 4.82, *p* = 0.003, partial η^2^ (i.e., the effect size) = 0.05, which was small. The *post hoc* (Bonferroni) tests revealed that participants with the education level of master's degree and above (*M* = 3.59, *SD* = 0.46) endorsed a higher degree of stigma toward recovered patients with COVID-19 than undergraduate participants (*M* = 3.37, *SD* = 0.30), *p* = 0.008. No significant differences existed among different subjective social classes on the COVID-19-related stigma, except that there were significant differences among different subjective social classes on stigma toward neighbors of recovered patients with COVID-19, *F*_(2,243)_ = 4.79, *p* = 0.009, partial η^2^ = 0.04, which was small. The *post hoc* (Bonferroni) tests revealed that participants who reported that their subjective social classes were 8–10 (*M* = 3.56, *SD* = 0.30) endorsed a higher level of stigma toward neighbors of recovered patients with COVID-19 than participants who reported that their subjective social classes were 4–7 (*M* = 3.36, *SD* = 0.29), *p* = 0.046.

**Table 8:** The differences between main variables regarding different individuals-related stigma toward COVID-19.

**Variables**		**RP (** ***n*** **=** **285)**		**RPFM (** ***n*** **=** **265)**		**RPN (** ***n*** **=** **246)**		**RPF (** ***n*** **=** **238)**		**FHP (** ***n*** **=** **120)**		**IHRA (** ***n*** **=** **306)**		**INRC (** ***n*** **=** **100)**	
		**Score *M* (*SD*)**	***t***	**Score *M* (*SD*)**	***t***	**Score *M* (*SD*)**	***t***	**Score *M* (*SD*)**	***t***	**Score *M* (*SD*)**	***t***	**Score *M* (*SD*)**	***t***	**Score *M* (*SD*)**	***t***
Gender	Male	3.44 (0.36)	1.60	3.40 (0.33)	1.30	3.43 (0.34)	2.19^*^	3.43 (0.33)	1.97	3.38 (0.28)	1.73	3.44 (0.36)	0.69	3.32 (0.26)	−0.76
	Female	3.37 (0.30)		3.35 (0.29)		3.35 (0.27)		3.35 (0.27)		3.30 (0.22)		3.41 (0.31)		3.36 (0.24)	
			*F*		*F*		*F*		*F*		*F*		*F*		*F*
Age	16–20	3.38 (0.32)	1.71	3.35 (0.29)	2.25	3.37 (0.31)	0.89	3.37 (0.30)	1.54	3.33 (0.25)	0.25	3.39 (0.30)	1.91	3.35 (0.26)	0.98
	21–30	3.35 (0.33)		3.46 (0.36)		3.45 (0.34)		3.46 (0.34)		3.37 (0.30)		3.49 (0.41)		3.31 (0.26)	
	31–40	3.49 (0.36)		3.36 (0.30)		3.36 (0.25)		3.34 (0.25)		3.38 (0.17)		3.52 (0.39)		3.19 (0.09)	
	41–54	3.48 (0.48)		3.24 (0.15)		3.28 (–)		3.29 (-)		– (–)		3.39 (0.22)		3.50 (–)	
Education Level	High school and below	3.61 (0.43)	4.82^**^	3.47 (0.46)	1.53	3.40 (0.45)	1.10	3.44 (0.46)	0.58	– (–)	2.37	3.55 (0.49)	2.37	– (–)	1.44
	College	3.46 (0.33)		3.40 (0.31)		3.40 (0.30)		3.40 (0.30)		3.40 (0.28)		3.42 (0.32)		3.40 (0.28)	
	Bachelor's degree	3.37 (0.30)		3.35 (0.29)		3.38 (0.29)		3.38 (0.29)		3.31 (0.23)		3.41 (0.32)		3.30 (0.24)	
	Master's degree and above	3.59 (0.46)		3.48 (0.43)		3.52 (0.43)		3.48 (0.42)		3.46 (0.40)		3.60 (0.46)		3.36 (0.30)	
Social Class	1–3	3.46 (0.37)	2.29	3.42 (0.35)	2.77	3.46 (0.37)	4.79^**^	3.44 (0.34)	2.42	3.44 (0.32)	2.90	3.49 (0.37)	1.40	3.42 (0.31)	1.47
	4–7	3.38 (0.32)		3.35 (0.30)		3.36 (0.29)		3.37 (0.30)		3.31 (0.22)		3.41 (0.33)		3.32 (0.23)	
	8–10	3.52 (0.31)		3.51 (0.29)		3.56 (0.30)		3.51 (0.31)		3.39 (0.28)		3.42 (0.29)		3.29 (0.27)	

*RP, recovered patients with COVID-19; RPFM, recovered COVID-19 patients' family members; RPN, recovered COVID-19 patients' neighbors; RPF, recovered COVID-19 patients' friends; FHP, frontline healthcare providers; IHRA, individuals in high-risk areas; INRC, individuals not related to COVID-19*.

* *p < 0.05*,

** *p < 0.01*.

## Discussion

### General Description of COVID-19-Related Stigma in China

Coronavirus disease 2019 has jeopardized human lives and societies worldwide. Resultantly, many researchers had conducted medical studies on COVID-19 (Ahn et al., 2020; Chakraborty et al., 2020; Landete et al., 2020; Wan et al., 2020). However, few researchers have conducted empirical studies regarding the COVID-19-related stigma, while various media reports suggested that stigma and discrimination cases related to COVID-19 were common globally, such as America, Nepal, Jordan, India, Italy, and China (Aacharya and Shah, 2020; Chopra and Arora, 2020; Khasawneh et al., 2020; Sahoo et al., 2020; Singh and Subedi, 2020; Turner-Musa et al., 2020). Thus, this study quantified the COVID-19-related stigma in China.

Fundamentally, H_1_ was supported. It was found that the prevalence of COVID-19-related stigma was low in China (14.84% of participants endorsed stigma toward recovered patients with COVID-19). One of the possible reasons is that the stigma belongs to a social issue, which means that the responses of participants in the questionnaires might be affected by the social desirability effect and concealed their true attitudes toward groups related to stigma. Thus, they might be particularly cautious when they chose options with scores >3 on the stigma scale, indicating that the sample of the stigma data >3 was small. However, the results in this study were worse than on SARS- and TB-related stigma in previous studies (Link and Phelan, 2001; Mak et al., 2006), which indicated that the percentage of participants who endorsed stigmatizing perceptions toward SARS and TB were 3.7 and 4.9%, respectively. This inconsistency might be because, during the COVID-19 outbreak, the regional lockdown programs had been enforced in China to block the possible chains of transmission wherein people were stayed at home to avoid contact with others, which conformed to the behavioral response of stigma (Mak et al., 2006). Moreover, this study showed that participants endorsed similar stigma toward families (13.80%), neighbors (12.81%), and friends (12.40%) of recovered patients with COVID-19 to participants endorsed stigma toward recovered patients. Furthermore, the distributions of COVID-19-related stigma toward recovered patients, and their families, neighbors, friends, and individuals in high-risk areas, had similar structures. The distributions of COVID-19-related stigma to frontline healthcare providers and individuals not related to COVID-19 were similar and had different types of structures. As expected, based on the BIS, one of its characteristics is overgeneralization, which indicates that the BIS has the tendency to be oversensitive or overgeneralized to cues related to diseases, even in cases where disease threats are absent (Ackerman et al., 2018). Just as the “smoke detector principle” (Nesse, 2005), all of the infectious or non-infectious psychical and mental abnormalities are regarded by the BIS as dangerous signals (Ackerman et al., 2018). Thus, although families, neighbors, and friends of recovered COVID-19 patients are not infected with COVID-19, the magnitude of the correlations between recovered patients and their families, neighbors, and friends is strong (*r* ranges from 0.92 to 0.99), which implied that they might have a higher probability of infection than others who are not related to recovered patients, and then the BIS may trigger psychological responses related to stigmatization, such as disgust and avoidance. These results uncovered that we should not only pay attention to patients with COVID-19 but also focus on their families, neighbors, and friends, who are likely to be ignored in the future.

This study unveiled that participants also reported a higher level of stigma toward individuals in high-risk areas (15.94%) than recovered patients with COVID-19 (14.84%). In addition to the effects of BIS on stigma as mentioned earlier, another possible reason for this may be that patients with COVID-19 who have recovered in this study were perceived by participants to be safer than the individuals in high-risk areas in which many people may be infected with the virus. Therefore, participants might be more afraid of individuals in high-risk areas than recovered patients with COVID-19 and be more likely to avoid them, which might eventually lead to greater stigma scores for individuals in high-risk areas. Additionally, during the COVID-19 pandemic, various media have reported incidents of the stigmatization of frontline healthcare providers worldwide, such as Mexico, Malawi, India, and the United States (Bagcchi, 2020; Grover et al., 2020). In this study, only 100 (5.21%) participants had stigmatizing perceptions of frontline healthcare providers. The reason may be that the traditional Chinese culture emphasizes “*do not forget what other people have done for you*” (Xu et al., 2018). With that mindset, frontline healthcare providers volunteered and exerted their best to provide healthcare for patients in China (Liu et al., 2020), influencing most Chinese to be grateful for them, instead of stigmatizing and discriminating against them. Future studies should investigate gratefulness (as well as stigma) in order to provide a fuller picture of the perception of healthcare providers. Additionally, future studies should investigate the stigma of individuals toward frontline healthcare providers in other cultural contexts.

To identify the possible factors that impact the COVID-19-related stigma, this study explored the relationship between the knowledge about COVID-19 and the COVID-19-related stigma. H_2_ was partially supported. The results showed that knowledge was negatively related to COVID-19-related stigma based on the whole dataset. Specifically, the less knowledge about COVID-19 participants had, the more COVID-19-related stigma they endorsed. However, after removing the data of stigma scores that were ≤3, this study found that no significant association existed between the knowledge about COVID-19 and the COVID-19-related stigma, which was inconsistent with previous studies (Svensson and Hansson, 2016; Lopez et al., 2018). Based on previous studies, a significantly negative relationship existed between knowledge and stigma. Given that the results of the whole stigma data differed from those of the stigma data >3, we further analyzed the relationship between the knowledge about COVID-19 and the stigma data ≤3. The results revealed that a significantly negative relationship existed between the knowledge about COVID-19 and the COVID-19-related stigma, which implied that the more knowledge about COVID-19 participants had, the less degree of stigmatization toward COVID-19 they endorsed. In summary, there might be a significantly negative relationship between the knowledge about COVID-19 and the COVID-19-related stigma, and the lack of significant findings on the subset of the data when the stigma scores were larger than 3 may be due to the limited range of scores. However, future studies should be cautious when using this conclusion.

Finally, this study explored whether demographic differences exist in COVID-19-related stigma. The results showed that gender, age, education level, and social class minimally affected the COVID-19-related stigma of individuals. This study found that participants with master's degree or higher education level endorsed higher degrees of stigma toward recovered patients with COVID-19 than undergraduate participants. In addition, participants who reported that their subjective social classes were 8–10 endorsed the higher levels of stigma toward neighbors of recovered patients with COVID-19 than participants who reported that their subjective social classes were 4–7. A higher subjective social class represents individuals who have higher incomes, degrees of education, and occupations. Thus, the two findings implied that people who have a higher level of education may hold a higher level of COVID-19-related stigma, which was inconsistent with the study of Johnco and Rapee (2018), which found that a negative association existed between education and stigma toward patients with diseases. Based on the abovementioned findings, given that a significantly negative relationship existed between the knowledge about COVID-19 and the degree of the COVID-19-related stigma, individuals who have a higher level of education might gain more knowledge about COVID-19 and should have endorsed less COVID-19-related stigma. Thus, we failed to explain the reasons for the results in this study. However, as Williams et al. (2011) mentioned, “disease-related stigma persists in spite of education and often without rationale” (p. 68), the relationship between disease-related stigma and education requires further investigation.

### Limitations

This study holds certain limitations. First, based on previous studies on stigma toward mental illness (Michaels and Corrigan, 2013; Latkin et al., 2017), the responses of participants in this study might be affected by the social desirability effect given that this study used questionnaires, which might influence the accuracy of the results. Therefore, to reduce the social desirability effect, the COVID-19-related stigma of participants should be investigated using indirect measures, such as implicit association tests (Greenwald et al., 1998). Second, 120 (6.25%) and 100 (5.21%) participants had stigmatizing perceptions toward individuals not related to COVID-19 and frontline healthcare providers, respectively. The results show the complexity of studying stigma. People somewhat have stigmas toward others. Thus, the values of 6.25 and 5.21% can be used as a baseline to evaluate the situations of COVID-19-related stigma in China. Future studies should pay attention to this baseline. Third, this study used the convenience sampling method, instead of the random sampling method. Most participants were university students, which leads to the uneven distribution of the sample by age and education. Therefore, the generalizability of the results may be limited, and the reliability of the results may be influenced. Future studies are suggested to investigate the COVID-19-related stigma based on children and elder individuals. Fourth, the recovered patients with COVID-19 and their families, friends, and neighbors were studied in this study. Moreover, whether the patients with COVID-19 undergoing rehabilitation and their families, friends, and neighbors experience more stigma should be studied given that they may be considered more dangerous than recovered patients with COVID-19 and their families, friends, and neighbors by other individuals. Furthermore, only two self-assessment questions were used to assess the degree of the knowledge of participants about COVID-19, which may not fully reflect the knowledge of participants about COVID-19 and may further cause the mixed results of the correlation between the knowledge about COVID-19 and its related stigma. Although there were studies measuring the knowledge of participants about medical health using the same two questions (e.g., Sørensen et al., 2012; Wang et al., 2018), future studies are suggested to use more questions and objective indicators to assess the knowledge of people about COVID-19. For example, the questions can be requiring participants to assess their knowledge about dissemination channels of transmission of COVID-19, such as from the air, animal, contaminated food, skin contact, and blood transfusion (Khasawneh et al., 2020). Finally, this study tested only the role of knowledge, but more potentially relevant individual characteristics exist that may impact COVID-19-related stigma in the pandemic. For example, Xu and Cheng (2021) indicated that political ideology, self-control, need for cognition, and risk attitude (health/safety risk-averse) were correlated with the attitudes of individuals toward COVID-19. Additionally, a core cognitive function, namely, working memory, was explored, and the working memory capacity predicted individual differences in social distancing compliance during the COVID-19 pandemic (Xie et al., 2020). Particularly, the higher working memory capacity that participants had, the more social distancing compliance they showed. Thus, these factors should be explored in the future.

Coronavirus disease 2019–related stigma has posed a serious threat to the psychical and mental health of individuals and the whole society. Therefore, we hope that the government, researchers, and general population pay attention to the treatment of COVID-19 itself while focusing on the negative social issues induced by the COVID-19-related stigma, especially its preventions and interventions (Stangl et al., 2019). For example, given that the COVID-19-related stigma is attributable to the unscientific belief and improper understanding of individuals (Bagcchi, 2020), it might be helpful for health professionals to recommend reliable and scientific information related to COVID-19 to expand the knowledge of individuals about COVID-19 to reduce or eliminate the COVID-19-related stigma.

## Conclusion

This study examined the COVID-19-related stigma in China. Ultimately, not only recovered patients with COVID-19 were susceptible to stigma, but also families, neighbors, friends of recovered patients with COVID-19, frontline healthcare providers, and individuals in high-risk areas. Moreover, there might be a significantly negative relationship between the knowledge about COVID-19 and the COVID-19-related stigma. Specifically, the knowledge about COVID-19 was negatively associated with the COVID-19-related stigma. A higher level of knowledge about COVID-19 might lead to a lower level of COVID-19-related stigma, although no significant relationship existed between the knowledge about COVID-19 and the COVID-19-related stigma in the groups who have held COVID-19-related stigma.

## Data Availability Statement

The original contributions presented in the study are included in the article/Supplementary Material, further inquiries can be directed to the corresponding author/s.

## Ethics Statement

Ethical review and approval was not required for the study on human participants in accordance with the local legislation and institutional requirements. Written informed consent from the participants' legal guardian/next of kin was not required to participate in this study in accordance with the national legislation and the institutional requirements.

## Author Contributions

LZ, ZW, and JG: conception and design. PS and ZW: administrative support. LZ, WZ, and PS: provision of study materials. ZW, LZ, GZ, and WZ: collection and assembly of data. LZ, ZW, and JG: data analysis and interpretation. All authors: manuscript writing and final approval of manuscript.

## Conflict of Interest

The authors declare that the research was conducted in the absence of any commercial or financial relationships that could be construed as a potential conflict of interest.

## Publisher's Note

All claims expressed in this article are solely those of the authors and do not necessarily represent those of their affiliated organizations, or those of the publisher, the editors and the reviewers. Any product that may be evaluated in this article, or claim that may be made by its manufacturer, is not guaranteed or endorsed by the publisher.
